# Transhiatal Esophagectomy after Previous Left Pneumonectomy: Challenge of Surgical Approach, a Case Report

**Published:** 2020-11

**Authors:** Abdoulhossein Davoodabadi, Mohammad Ali Saba, Abbas Arj, Hamidreza Talari

**Affiliations:** 1 Department of Surgery, Kashan University of Medical Sciences (KAUMS), Kashan, Iran,; 2 Department of Pulmonology, Kashan University of Medical Sciences (KAUMS), Kashan, Iran,; 3 Department of Gastroenterology, Kashan University of Medical Sciences (KAUMS), Kashan, Iran,; 4 Department of Radiology, Kashan University of Medical Sciences (KAUMS), Kashan, Iran.

**Keywords:** Esophageal cancer, Lung cancer, Pneumonectomy, Transhiatal esophagectomy

## Abstract

The occurrence of esophageal cancer after previous pneumonectomy for primary lung cancer is rare. This is the second case report of transhiatal esophagectomy after previous pneumonectomy due to lung cancer. In this case, selection of surgical approach for esophagectomy, was technically challenging and anatomic deformity in post pneumonectomy space had potential risk of physiologic disturbance, especially after thoracotomy option in solitary lung with limited capacity.

**Case presentation:**

We herein report a 58 year old man with history of left pneumonectomy and lymph node dissection due to mucoepidermoid carcinoma 19 years ago and recently admitted for esophageal carcinoma. He successfully was managed via transhiatal approach.

**Conclusion:**

Transhiatal esophagectomy in pneuminectomized patient is safe and recommended as first option.

## INTRODUCTION

Esophagectomy in setting of prior pneumonectomy is a challenging issue, and there is little experience in this area with only a few cases reported to date (1–5). This is the second report of a transhiatal esophagectomy after prior pneumonectomy due to lung cancer.

## CASE SUMMARY

A 58-year-old man referred to our teaching hospital with progressive dysphagia from 2 months ago. He could only take down soft drink. The patient had undergone left pneumonectomy and lymph node dissection for mucoepidermoid carcinoma (pT3N1M0) 19 years ago. He was worker with no history of smoking but recently became diabetic and was controlled with oral anti hyperglycemic drugs. He underwent barium swallow which showed irregular mass and deformity in lower third, then Esophagoscopy showed tumor in 32 cm from the incisors. Biopsies were taken and moderately differentiated squamous cell carcinoma was the confirmed diagnosis.

Preoperative spiral chest and abdominal CT scan was done to evaluate the thoracic anatomy, lymphadenopathy and surgical plan and approaches. Whole body scan was done to assess the presence of any metastatic disease.

**Figure 1.**
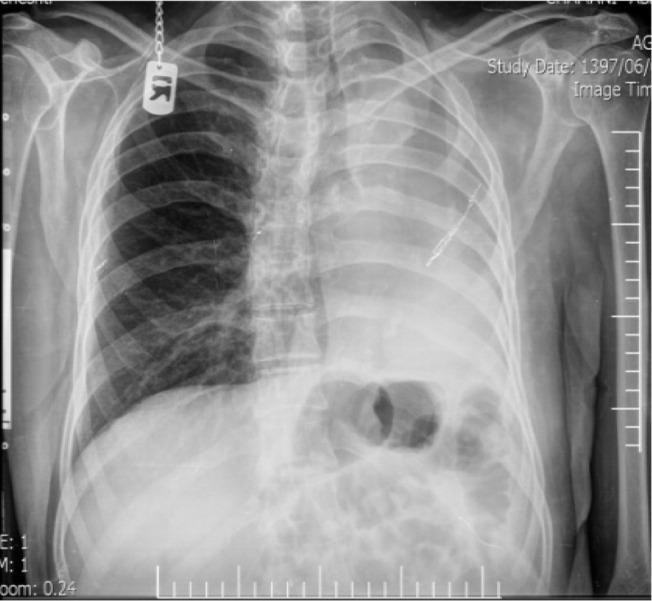
Chest x ray, anatomic deviation after left pneumonectomy

**Figure 2.**
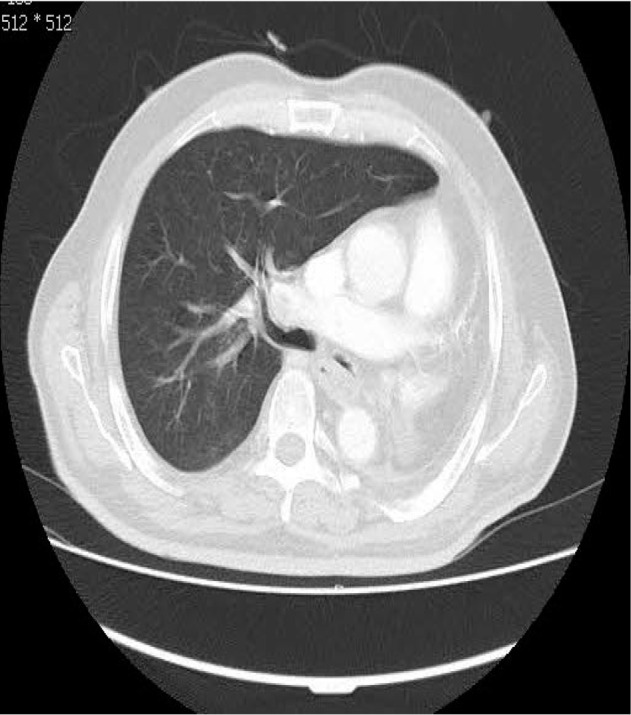
Axial lung window CT scan, anatomic deviation after left pneumonectomy

CT of the chest demonstrated marked anatomic deviation, hyper expansion of the right lung, mediastinum shift to the left hemithorax, and reduced left intrathoracic space with heterogeneous opacification (Figure 1, 2). No metastasis or lymphadenopathy was found in brain magnetic resonance imaging (MRI), whole body scan, chest and abdominal CT scan.

**Figure 3.**
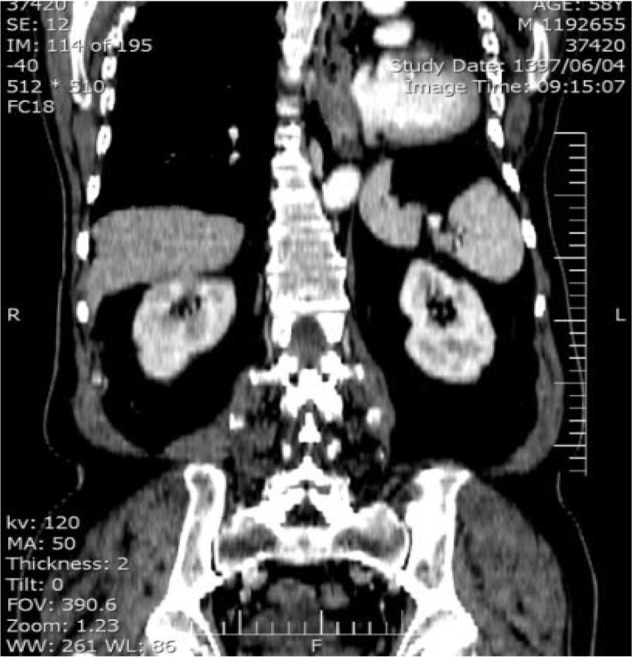
Frontal CT scan, marked deviation after left pneumonectomy.

In preoperative cardiovascular workup, ejection fraction was 60%, Pulmonary function test also showed a forced expiratory volume (FEV1) of 1.23 L (56% predicted), forced vital capacity (FVC) of 1.50 L (50% predicted). ABG in room atmosphere was normal and he could walk two floors up without dyspnea.

Our assessment was based on the preoperative cardiovascular assessment, pulmonary function tests, data and imaging indicating the operability of tumor. The preferred method was an approach without thoracotomy due to limited lung capacity and possible physiologic disturbance. Thus, the initial operative plan was transhiatal approach. However, possible intra operative unpredictive findings may necessitate change the operative approach. Although transthoracic tree hole approach was an alternative favorite, operating in solitary lung and manipulation in this area may be hazardous. We had many difficulties in entering the left chest, due to extensive fibrotic scar tissue, obliteration of the post pneumonectomy space and significant anatomic distortion, so, the final decision was made to precede on Transhiatal esophageal resection without any neo adjuvant modality.

### Informed consent

A writhen informed consent was obtained from the patient and his relatives for possible major complications, high morbidity and mortality.

### Procedure

Preparation of the colon was performed earlier. After careful preparation in well-equipped operating room, full monitoring and insertion of 2 intra venous line for immediate blood transfusion, the patient underwent laparotomy with upper midline incision. On abdominal exploration, no evidence of metastatic disease was seen. Esophageal hiatus mobilization and conduit preparation were performed in the standard fashion as: proximal duodenum and stomach with vascular pedicles were mobilized, gastric conduit for esophageal substitute on base of the right gastroepiploic and right gastric artery with cutting linear GI staple were prepared, and pyloromyotomy was performed.

We encountered relative dense adhesions at the hiatus and distal of esophagus that extended into the left hemithorax. Esophageal hiatus was widened as much as possible to facilitate more access for paraesophageal tissue and more controlling of the vital structures.

To facilitate adequate length for the cervical anastomosis, we performed a more extensive Kocher maneuver to allow greater conduit mobilization and avoiding possible colonic bypass.

With careful dissection in posterior mediastinum and paraesophageal tissue, distal of tumor about 5 cm above gastro esophageal junction (GEJ) was palpable, and dissection continued into proximal tumor meticulously. It seemed dissection of paraesophageal tissue was feasible, not hazardous. Continuing dissection was promising and safe and confirmed our initial impression that blunt mobilization would be possible.

Then left sided cervical incision from the sternal notch to the level of the cricoid cartilage medial to sternocleidomastoid was done, cervical esophagus was dissected from adjacent tissue carefully, dissection was continued in to upper thorax, the esophagus was mobilized from the trachea, great vessels and thick fibrous pleura and the mediastinal contents. Blunt dissection of esophagus from upper thorax and lower hiatus was performed simultaneously. Then the mobilized esophagus above sternal notch was cut, for removal of esophagus a Nelaton tube was inserted into the lumen and proximal portion of the tube circumferentially sutured to proximal edge of esophagus and extracted in to abdominal cavity.

Because of dense adhesions we were hardly able to release the periesophageal tissue; however, our final specimen was stripped adventitial tissue.

Then gastric conduit was brought up to the neck in a sterile manner and functional end-to-end anastomosis with separated absorbable monofilament suture was performed. A nasal gastric tube was inserted into the gastric conduit and pushed several centimeter after pyloromyotomy site as feeding tube. During operation, no adverse event and hemorrhage hemodynamic instability happened; O_2_ saturation was optimized and blood lost was near 500 cc.

**Figure 4.**
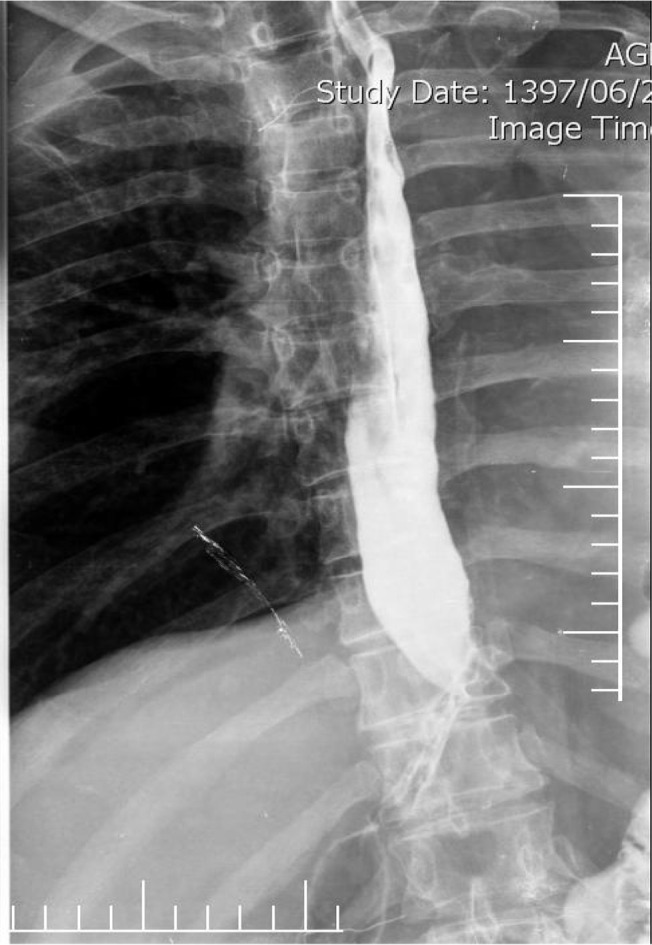
Thin barium esophagogram

The patient’s postoperative course in ICU was well managed. The next day he was extubated without any respiratory distress and complication. On the 3rd day after operation, warm tea, and then soft diet via NG tube was started, on 5th day patient was transferred to ward and on 9th day thin barium esophagogram showed good position of the new esophageal conduit, no evidence of a leak and normal emptying of the conduit (Figure 4). He tolerated regular diet and was discharged on 10th postoperative day, to be referred to oncologist. Final pathology revealed primary moderately squamous cell carcinoma with only adventitia involvement (pT3N0M0). The patient during 3-month follow-up was active, well, did not have any complications and his voice was intact without aspiration or dysphagia.

## DISCUSSION

There is dilemma regarding the approaching side, as pneumonectomy has left substantial deformity in the ipsilateral thoracic space, whereas operating on the healthy side puts the lung at risk of respiratory difficulty (6). Following pneumonectomy, the ipsilateral hemithorax was contracted and repeated entry into the thoracic cavity presented several challenges, as a result of postoperative compensatory anatomic and physiologic changes such as mediastinal shift toward the operated side, elevation of the ipsilateral hemidiaphragm, and hyper expansion of the remaining lung (7,8).

Esophagectomy through the right thoracotomy in patient that had undergone left pneumonectomy, would carry more complication, such as the potential pleural space contamination.

Transhiatal esophagectomy is theoretically appealing in avoiding the thoracotomy but technically may not be feasible due to post pneumonectomy changes, and previous mediastinal lymph node dissection (1). However, it may be considered as one the best promising approach that could be used to avoid serious problems in thoracic entry.

The principles of transhiatal esophagectomy in post pneumonectomy patients are the same as conventional, however it requires preparation of the colon as alternative substitute, more caution in tissue dissection, well equipped operating room, full monitoring, and availability of immediate blood transfusion. Also hiatus must be widened as much as possible for better accessibility of vital paraesophageal structures, allowing simultaneous use of laparoscope for adequate exposure and lysis of adhesions (1).

In this patient neoadjuvant radiation therapy is not recommended because of concern of worsening of extensive fibrosis that is already present from previous pneumonectomy, although data this area is not adequate. One the most important reasons of selecting transhiatal approach was our greater experience in transhiatal esophagectomy than any others option and many of surgeons may be unfamiliar with post pneumonectomy area with many pitfalls. Moreover, transhiatal esophagectomy may protect the residual pulmonary reserve, managing the potential anastomotic leak better.

Although our experience in this case revealed transhiatal esophagectomy in pneuminectomized patient is safe, and may be recommended as first option, but this surgical approach should be optimized on individual basis. The procedure presented here is applicable for tumors of the lower third, while tumors located in the upper and middle third may be impossible for resection with this approach; however, extracorporeal membrane oxygenation (ECMO) has been introduced to enable surgery in these patients (4).

## CONCLUSION

In pneumonectomized patient, transhiatal approach for resection of lower third esophagus cancer is safe and may recommended as first option; however, other safe options individually must to be considered.
